# Umbilical Cord Mesenchymal Stromal/Stem Cells and Their Interplay with Th-17 Cell Response Pathway

**DOI:** 10.3390/cells13020169

**Published:** 2024-01-16

**Authors:** Mehdi Najar, Saida Rahmani, Wissam H. Faour, Sami G. Alsabri, Catherine A. Lombard, Hussein Fayyad-Kazan, Etienne M. Sokal, Makram Merimi, Hassan Fahmi

**Affiliations:** 1 Osteoarthritis Research Unit, Department of Medicine, University of Montreal Hospital Research Center (CRCHUM), Montreal, QC H2X 0A9, Canada; 2 Faculty of Medicine, Université Libre de Bruxelles, 1070 Brussels, Belgium; 3 LBBES Laboratory, Genetics and Immune Cell Therapy Unit, Faculty of Sciences, University Mohammed Premier, Oujda 60000, Morocco; 4 Gilbert and Rose-Marie Chagoury School of Medicine, Lebanese American University, P.O. Box 36, Byblos 5053, Lebanon; 5 Laboratory of Pediatric Hepatology and Cell Therapy, Institut de Recherche Expérimentale et Clinique (IREC), Université Catholique de Louvain, 1200 Brussels, Belgium; 6 Laboratory of Cancer Biology and Molecular Immunology, Faculty of Sciences-I, Lebanese University, P.O. Box 6573/14, Beirut 1103, Lebanon

**Keywords:** UC-MSCs, Th-17 cells, inflammation, immunomodulation, tissue repair

## Abstract

As a form of immunomodulatory therapeutics, mesenchymal stromal/stem cells (MSCs) from umbilical cord (UC) tissue were assessed for their dynamic interplay with the Th-17 immune response pathway. UC-MSCs were able to modulate lymphocyte response by promoting a Th-17-like profile. Such modulation depended on the cell ratio of the cocultures as well as the presence of an inflammatory setting underlying their plasticity. UC-MSCs significantly increased the expression of IL-17A and RORγt but differentially modulated T cell expression of IL-23R. In parallel, the secretion profile of the fifteen factors (IL1β, IL-4, IL-6, IL-10, IL-17A, IL-17F, IL-22, IL-21, IL-23, IL-25, IL-31, IL-33, INF-γ, sCD40, and TNF-α) involved in the Th-17 immune response pathway was substantially altered during these cocultures. The modulation of these factors demonstrates the capacity of UC-MSCs to sense and actively respond to tissue challenges. Protein network and functional enrichment analysis indicated that several biological processes, molecular functions, and cellular components linked to distinct Th-17 signaling interactions are involved in several trophic, inflammatory, and immune network responses. These immunological changes and interactions with the Th-17 pathway are likely critical to tissue healing and may help to identify molecular targets that will improve therapeutic strategies involving UC-MSCs.

## 1. Introduction

Umbilical cord mesenchymal stromal/stem cells (UC-MSCs) represent an important therapeutic cellular product due to their interesting medicinal properties. Compared to other sources, the UC shows significant advantages over other sources regarding MSC isolation. They can be sourced from various areas of the umbilical cord, including the Wharton’s Jelly tissue. As a commonly discarded tissue, the umbilical cord contains a rich source of MSCs with a high cell yield and therefore is obtained noninvasively and with no ethical issues [1]. The morphology, immunophenotype, proliferation, multidirectional differentiation, and ability to support hematopoiesis are mostly similar to those from other sources, but UC-MSCs have higher immunomodulatory capacities and low immunogenicity [2]. According to the International Society for Cellular Therapy (ISCT), MSCs present in vitro a fibroblast-like shape with plastic adherence capacity and the potential to differentiate into distinct cellular lineages and specific immunophenotypes (expression of CD73, CD90, CD105, and lack of CD14, CD19, CD34, CD45, and HLA-DR) [3].

The wound healing process is the final step to restore tissue integrity following injury. The mechanisms and controls of wound healing (inflammation, etc.) are complex and involve collaborative efforts of many cell types (fibroblasts, immune cells, etc.) and several factors (inflammatory mediators, growth factors, cytokines, and antimicrobial peptides) produced at the lesion site [4]. MSCs are able to migrate toward the sites of injury and inflammation, where they display their functions. Based on accumulated evidence from preclinical models, MSCs exert their therapeutic effects following reprogramming by the host injury microenvironment [5]. The main therapeutic mechanism of MSCs is to sustain the generation of a pro-regenerative and immunotolerogenic microenvironment allowing graft acceptance and tissue repair. MSCs establish crosstalk with both resident and infiltrating immune cells to maintain tissue homeostasis. MSCs may either influence target immune cells directly or indirectly by affecting the activities of other immune cells. Innate immunity provides the initial host response to tissue injury, trauma, and pathogens. Innate immunity activates adaptive immunity, and both act together in a highly orchestrated manner to establish and maintain tissue homeostasis [6]. Any dysregulation of this interaction can result in chronic inflammation and autoimmunity and is thought to be a major limiting setback for graft acceptance and may therefore impact the functions of MSCs. The secretome of MSCs represents the major way by which MSCs function and rely on the release of several regulatory molecules. However, the functions of MSCs are significantly influenced by the surrounding molecular and cellular microenvironment and respond adequately by adjusting their regulatory machinery. Interestingly, MSCs are considered sensors and switchers of inflammation [7], which plays a vital role in both normal and pathological healing [8]. Inflammation is a biological response of the immune system that can be triggered by a variety of factors, including pathogens, damaged cells, and toxic compounds, potentially leading to tissue damage or disease [9]. Despite encouraging results (safety concerns), conclusions from clinical trials using MSCs in inflammatory/immune contexts suggest that consistent and robust clinical improvement still needs to be reached. It is important to note that not all patients respond to treatment, indicating a need for further understanding of their reactivity with the immune system and mechanisms of action [10]. Indeed, the cell therapy outcome is likely conditioned by the patient’s immunological status and the tissue healing process. Upon tissue damage, several cell types are necessary to ensure a prompt healing process. Inflammation is an early event regulated by both innate and adaptive immune cell populations, including T helper-17 cells (Th-17 cells) [11]. Transcription factors compete to generate and specify the full Th-17 program, suggesting a higher plasticity potential and thus the possibility of interconversion between different T helper subsets [12,13]. The involvement of Th-17 cells in wound healing and the role of their associated cytokines is discussed, and both pro-reparative effects and deleterious effects are reported if not well regulated in some cases [11].

Th-17 cells and Th-17-cell-associated cytokines can promote inflammatory responses and impact wound healing [11]. Th-17 cells produce various cytokines, promoting cell survival, proliferation, and thus tissue regeneration. They are also able to convert from a proinflammatory phenotype to an anti-inflammatory one by changing their cytokine expression pattern, thereby limiting their own pathological potential [11].

Clarifying the communication of UC-MSCs and the local immune response during tissue healing processes is required to achieve optimal regenerative effects. Thus, it is important to understand the interaction between Th-17 cells and UC-MSCs for tissue repair purposes. Profiling the type of lymphocytes present during coculture with MSCs and analyzing the impact of the cell ratio and inflammation signals on such interactions is needed for an efficient effect. The changes within the secretome of such a coculture should also be determined. We observed that a Th-17-like profile arises from the coculture of MSCs with T cells. The Th-17 pathway seems to be promoted by MSCs in a cell-ratio- and inflammatory-dependent manner. The expression of Th-17-specific markers, including interleukin-17A, interleukin-23 receptor (IL-23R), and retinoic-acid-related orphan receptor gamma t (RORγt), as determined by flow cytometry, was differentially altered. Moreover, UC-MSCs significantly modified the secretome with the coculture system mainly by changing the secretion profile and levels of Th-17 pathway-related cytokines (IL-1β, IL-4, IL-6, IL-10, IL-17A, IL-17F, IL-21, IL-22, IL-23, IL-25, IL-31, IL-33, INF-γ, sCD40, and TNF-α). UC-MSCs are likely sensitive and responsive to inflammation and display functional plasticity, which allows interaction and therefore modulates lymphocyte differentiation in a dose-dependent manner. Th-17 cells and their associated cytokines can impact wound healing positively by clearing pathogens and can promote cell survival and proliferation and thus tissue regeneration [11].

Taken together, the UC might set the path for the development of new therapeutic applications in personalized regenerative medicine. The crosstalk between MSCs and Th-17 cells or their associated cytokines in an inflammatory setting is likely more complex than previously thought. These observations are the first to evaluate the behavior of UC-MSCs in the presence of lymphocytes with a Th-17-like profile. The local inflammatory response sustained by Th-17 cells and Th-17-cell-associated cytokines may modulate the tissue healing process within the injured tissue. This may be relevant to understanding the therapeutic processes of UC-MSCs in inflammatory conditions and to screening the signals that strengthen or hamper their functions. The upregulation of cytokines and regulatory mediators demonstrates the great capacity of UC-MSCs to sense and actively respond to tissue challenges. The inflammatory phase is important for tissue regeneration due to the release of proinflammatory cytokines and growth factors from immune cells [11]. The presence of inflammation within the patient during the use of MSCs may modulate their immunoregulatory activity and therefore influence therapeutic outcomes. The next critical issue resides in being able to select the most permissive cell product for personalized therapeutic benefit according to each patient’s characteristics. Functional enrichment analysis demonstrated that several biological processes, molecular functions, and cellular components linked to the Th-17 pathway are involved in complex trophic, inflammatory, and immune response networks. Approaches to identify specific markers or signatures associated with therapeutic benefits should be explored to identify responder and nonresponder patients. Artificial intelligence and deep-learning models could be trained to predict therapeutic MSC efficacy in a given pathologic background, such as inflammatory settings.

## 2. Materials and Methods

### 2.1. Isolation and Culture of UC-MSCs

This study was conducted in accordance with the Declaration of Helsinki (1964) and approved by the local ethics committee of the “Institut Jules Bordet” (Belgium). Umbilical cords (*n* = 5) were obtained from healthy pregnant mothers after full-term delivery with informed written consent. UCs were directly processed according to our previously described protocol [14]. Briefly, cords were transferred to aseptic saline buffer, washed with HBSS (Lonza, Basel, Switzerland), and cut into small pieces. The samples were sectioned longitudinally and completely immersed in Dulbecco’s modified Eagle’s medium with low glucose (Lonza: DMEM-LG) supplemented with 10% FBS (Sigma-Aldrich, St. Louis, MO, USA), 2 mmol/L l-glutamine, and 50 U/mL penicillin/streptomycin (Lonza). After 15 days, UC fragments were removed from the well to allow cell culture to begin. When subconfluence (80–90%) was achieved, adherent cells were harvested using TrypLE Select (Gibco, Life Technologies, Carlsbad, CA, USA) and expanded until the desired passage. Cell cultures were incubated at 37 °C in a 5% CO_2_ humidified atmosphere. After 48 h, nonadherent cells were removed by washing, and the medium was changed twice a week.

### 2.2. Phenotype of UC-MSCs

The phenotype of UC-MSCs was determined by flow cytometry using a panel of monoclonal antibodies (Table 1). Adherent cells were harvested with TrypLE Select (Lonza) solution, washed by centrifugation in PBS (Lonza), and finally resuspended in Miltenyi Biotec buffer. Then, harvested cells were incubated for 30 min at room temperature (RT) with conjugated primary antibody. After the labeling period, the cells were again washed, resuspended in PBS, and fixed with 4% paraformaldehyde.

### 2.3. In Vitro Trilineage Potential of UC-MSCs

The trilineage potential of UC-MSCs was confirmed by inducing adipogenic, osteogenic, and chondrogenic lineage commitment using specific culture conditions (NH media, Miltenyi Biotec, Leiden, The Netherlands) as previously described [15].

#### 2.3.1. Osteogenic Differentiation

A total of 5000 cells/well were plated in a 24-well plate with classical culture medium. After 5 days, the medium was replaced by osteogenic medium (StemMACS Osteo-Diff Media, Miltenyi Biotec). Cells were fed weekly with complete replacement of osteogenic medium. After 21 days, the mineralization of the extracellular matrix was assessed by Alizarin red staining. The cells were cleaned with phosphate-buffered saline (PBS) and then treated with 70% ethanol at room temperature for 5 min. Afterward, they were rinsed with H_2_O multiple times. Cells were stained with 40 mM Alizarin red (Sigma-Aldrich) at pH = 4.2 for 15 min at room temperature, rinsed in H_2_O, and then air-dried. Red staining was examined using light microscopy.

#### 2.3.2. Adipogenic Differentiation

A total of 5000 cells/well were plated in a 24-well plate with classical culture medium. After 5 days, the medium was replaced with adipogenic medium (StemMACS AdipoDiff Media, Miltenyi Biotec). Cells were fed weekly with complete replacement of adipogenic medium. On Day 7, the cells were stained with Oil Red O solution (Sigma) after fixation (8% formaldehyde). Lipid vacuoles were then observed by light microscopy.

#### 2.3.3. Chondrogenic Differentiation

A total of 150,000 cells were cultured in the tip of a 15 mL conical tube (Greiner, Kremsmünster, Austria) to enable cell culture in micromass with chondrogenic medium (Stem MACS Chondro Diff Media, Miltenyi Biotec). Cells were resuspended carefully and cultured at 37 °C in a 5% CO_2_ humidified atmosphere with the cap slightly screwed. Half of the chondrogenic medium was replaced weekly. On Day 21, aggregates were stained with Alcian blue (Sigma) to highlight cartilage proteoglycans. In some cases, cryosectioned pellets were stained with Alcian blue to confirm chondrogenic differentiation.

### 2.4. Inflammatory Preconditioning of UC-MSCs

UC-MSCs were preconditioned overnight using a cocktail of proinflammatory cytokines (25 ng/mL IL-1β, 10^3^ U/mL IFN-γ, 50 ng/mL TNF-α, and 3 × 10^3^ U/mL IFN-α) as previously performed [16].

### 2.5. Immune Cells

Peripheral blood (PB) samples were collected from healthy adult donors after signing an informed consent form. Briefly, peripheral blood mononuclear cells (PBMCs) were isolated using Ficoll-Hypaque gradient centrifugation of peripheral blood. Purification of total CD3^+^ T lymphocytes (>95% purity) was performed by positive selection using the MACS system (Miltenyi Biotec). T cells were activated by using a cocktail of mitogenic stimuli [phytohemagglutinin (PHA, 5 μg/mL; Remel) and interleukin-2 (IL-2, 20 U/mL; Biotest AG)].

### 2.6. Coculture of Immune Cells with UC-MSCs

To perform the coculture assay, preconditioned or nonpreconditioned UC-MSCs obtained after two passages (P2) were plated at 5 × 10^4^ cells/cm^2^ in a flat-bottomed 24-well plate. After overnight adherence, stimulated CD3^+^ T cells were incubated with plated UC-MSCs at cell ratios of 1/80 and 1/5 for 5 days in RPMI-1640 medium supplemented with 10% FBS.

### 2.7. Morphology of Cocultured Cell Populations

The coculture and morphological shapes of MSCs and T cells during coculture were assessed by phase contrast microscopy (100×) using a fluorescence inverted microscope (Leica, Machelen, Belgium).

### 2.8. IL-17A Expression

IL-17A expression in CD3^+^ T cells was determined by flow cytometry using a human IL-17A APC-conjugated monoclonal antibody based on the protocol provided by R&D Biosystems.

### 2.9. RORγt Expression

RORγt expression in CD3^+^ T cells was determined by flow cytometry using a human/mouse RORγt/RORC2/NR1F3 monoclonal antibody based on the protocol provided by R&D Biosystems.

### 2.10. IL-23 Receptor Expression

IL-23 receptor expression in CD3^+^ T cells was determined by flow cytometry using a human IL-23R PE-conjugated monoclonal antibody based on the protocol provided by R&D Biosystems.

### 2.11. Cytokine Secretion Profile

Supernatants from various culture conditions were collected and stored at −20 °C to analyze cytokines implicated in the Th-17 cell immune response pathway. The Bio-Plex ProTM Human Th-17 Cytokine Assays^®^ (Bio-Rad Laboratories, Inc., Hercules, CA, USA) were used to evaluate the following cytokines (IL-1β, IL-4, IL-6, IL-10, IL-17A, IL-17F, IL-21, IL-22, IL-23, IL-25, IL-31, IL-33, IFN-γ, soluble CD40 ligand (sCD40L), and TNF-α) according to the manufacturer’s recommendations (Bio-Rad Laboratories, Inc., Hercules, CA, USA).

### 2.12. Flow Cytometry

After cell staining, the data were acquired and analyzed using a MacsQuant analyzer (Miltenyi Biotec, Leiden, The Netherlands). The percentage (%) of positive UC-MSCs expressing a given marker is presented.

### 2.13. Construction and Analysis of the Protein–Protein Interaction Network

The PPI network was predicted using the Search Tool for the Retrieval of Interacting Genes (STRING) online database ([17]; version 11.0) (accessed on 14 August 2023). The STRING database (which relies on many data sources) was also used for the functional annotation and pathway enrichment analysis.

### 2.14. Statistical Analysis

Data are expressed as the mean ± standard error of the mean. For paired samples, statistical analysis was carried out using Prism software (GraphPad Prism 7.00, La Jolla, CA, USA). For repeated measurements, ANOVA was used to determine statistical significance, followed by a Newman-Keuls multiple comparison test. Values of * *p* ≤ 0.05, ** *p* ≤ 0.01, or *** *p* ≤ 0.001 were judged statistically significant.

## 3. Results

### 3.1. Characterization of UC-MSCs

#### 3.1.1. The Phenotype of UC-MSCs

**Figure 1:**
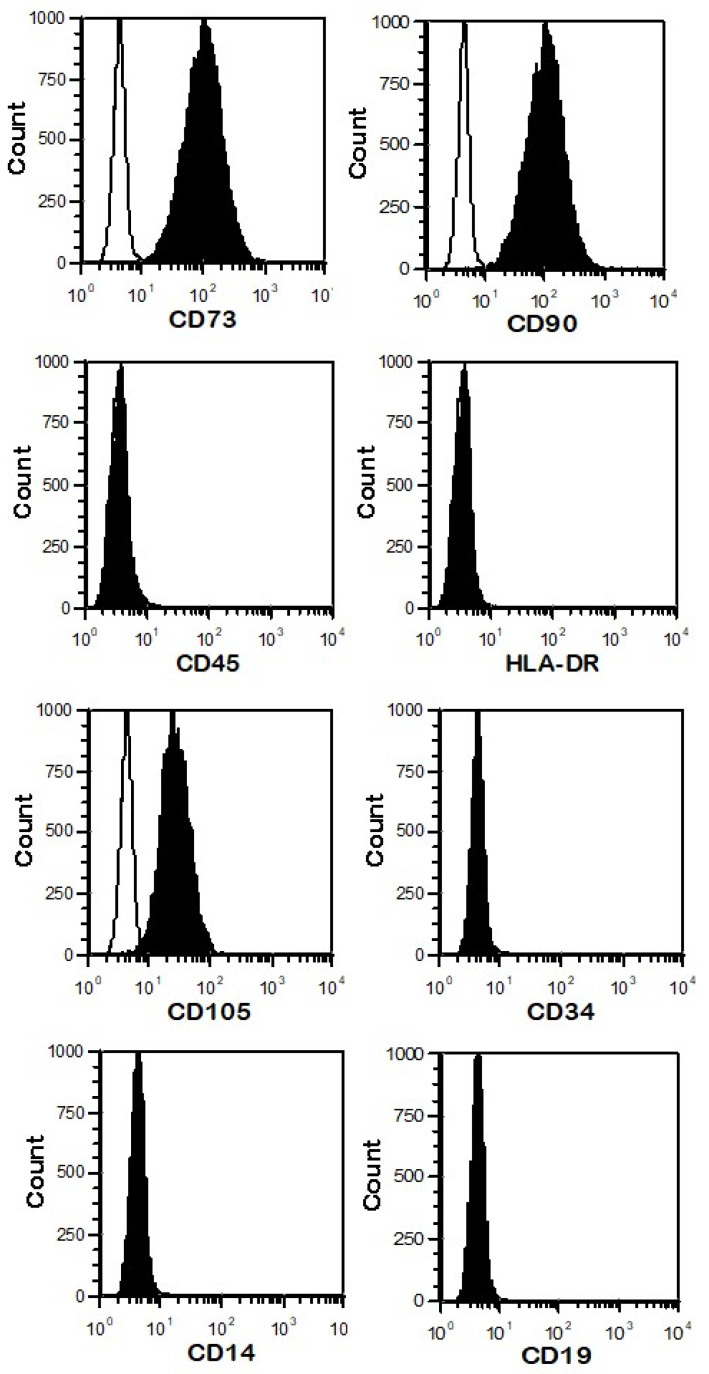
The phenotype of UC-MSCs. UC-MSCs (*n* = 5) were stained with fluorochrome-labeled monoclonal antibodies against CD73, CD90, CD45, HLA-DR, CD105, CD34, CD14, and CD19 (full histograms) and analyzed by flow cytometry. Empty histograms in each picture reflect background staining with the isotype control (control staining), whereas solid-black histograms represent specific staining for the relevant cell surface markers. The marker for each analysis is provided beneath the graphic.

The phenotype of UC-MSCs was analyzed based on the International Society for Cellular Therapy (ISCT) criteria. Such a profile was abundantly documented in our previous publications as a part of quality control assay [16]. A representative flow cytometry profile based on our previous research is shown in Figure 1. UC-MSCs expressed CD73 (98.75 ± 0.95%), CD90 (99.20 ± 0.61%), and CD105 (97.90 ± 0.69%), but they did not express CD45 (1.45 ± 0.55%), CD14 (1.30 ± 0.39%), CD19 (1.05 ± 0.42%), CD34 (2.05 ± 0.75%), and HLA-DR (1.10 ± 0.25%) markers.

#### 3.1.2. In Vitro Trilineage Potential of UC-MSCs

**Figure 2:**
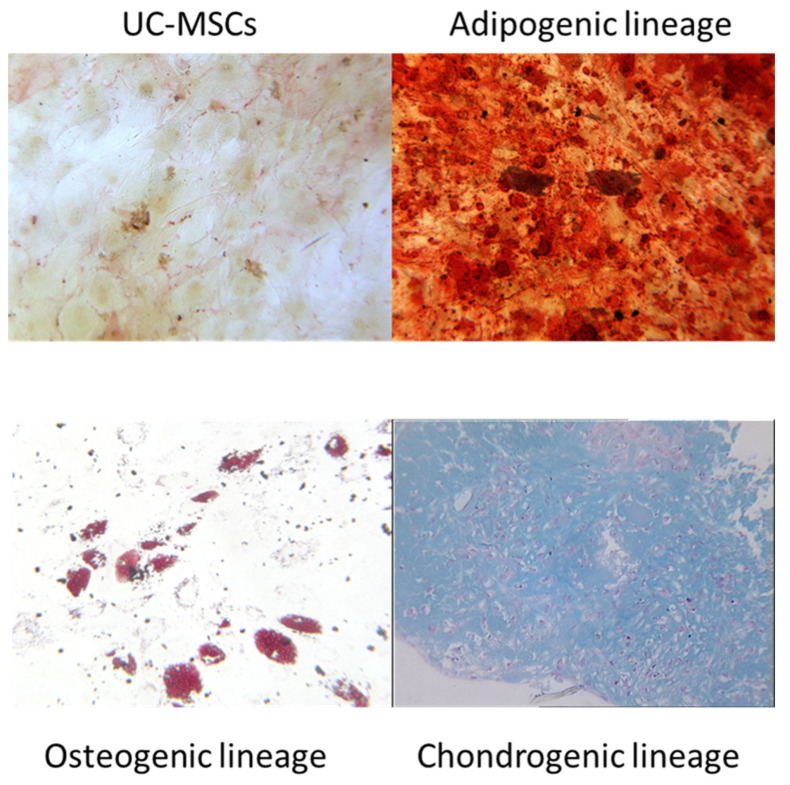
In vitro trilineage potential of UC-MSCs. The in vitro differentiation profile of UC-MSCs (*n* = 5) was determined following specific induction culture and staining techniques (magnification 100×). A representative example of an osteogenic lineage commitment was evaluated after 21 days of induction by Alizarin red staining to reveal mineralization. A representative example of adipogenic lineage commitment was evaluated after 7 days of induction by Oil Red O staining to detect lipid-containing vacuoles. A representative example of chondrogenic lineage commitment was evaluated after 21 days of induction by Alcian blue staining to color the presence of sulfated proteoglycan-rich extracellular matrix.

The in vitro trilineage potential of UC-MSCs was investigated using a specific induction medium and highlighted by using lineage-specific cell-staining techniques and microscopic examination. After 21 days of osteogenic differentiation, osteoblasts were observed, and matrix mineralization after calcium deposition was confirmed by Alizarin red staining. After 7 days of adipogenic differentiation, adipocytes were identified with cytoplasm containing a high number of small vacuoles with lipid accumulation revealed by Oil Red O staining. After 21 days of chondrogenic differentiation, cell pellets of 1–2 mm diameter were observed, and the production of a proteoglycan-based extracellular matrix was evidenced by Alcian blue staining of histological sections. These issues were abundantly documented in our previous publications as a part of a quality control assay [18]. Figure 2 presents a representative microscopic staining examination based on our previous research.

#### 3.1.3. Morphology of Cocultured Cell Populations

**Figure 3:**
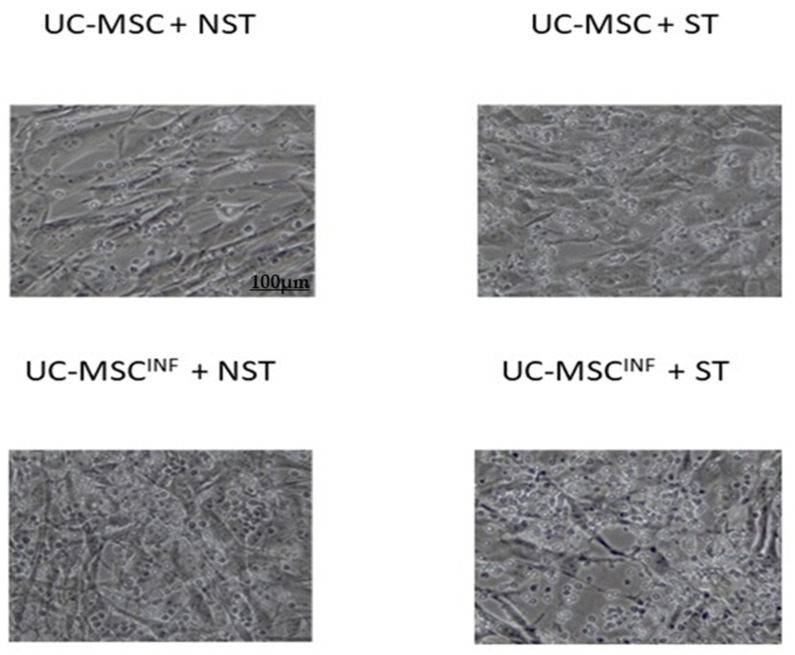
Contrast microscopic representation of UC-MSC (*n* = 5) cocultures with T cells after 5 days. UC-MSCs: umbilical cord mesenchymal stromal/stem cells; UC-MSCs^INF^: preconditioned umbilical cord mesenchymal stromal/stem cells; NST: unstimulated T cells; ST: PHA/IL-2-stimulated T cells.

UC-MSCs and T cells both preserved their morphology as fibroblast and round-like shapes, respectively (Figure 3). During coculture, there were clear and strong interactions between both populations, as observed by the adherence of T cells to UC-MSCs.

### 3.2. Expression of IL-17A in T Cells during Coculture with UC-MSCs

**Figure 4:**
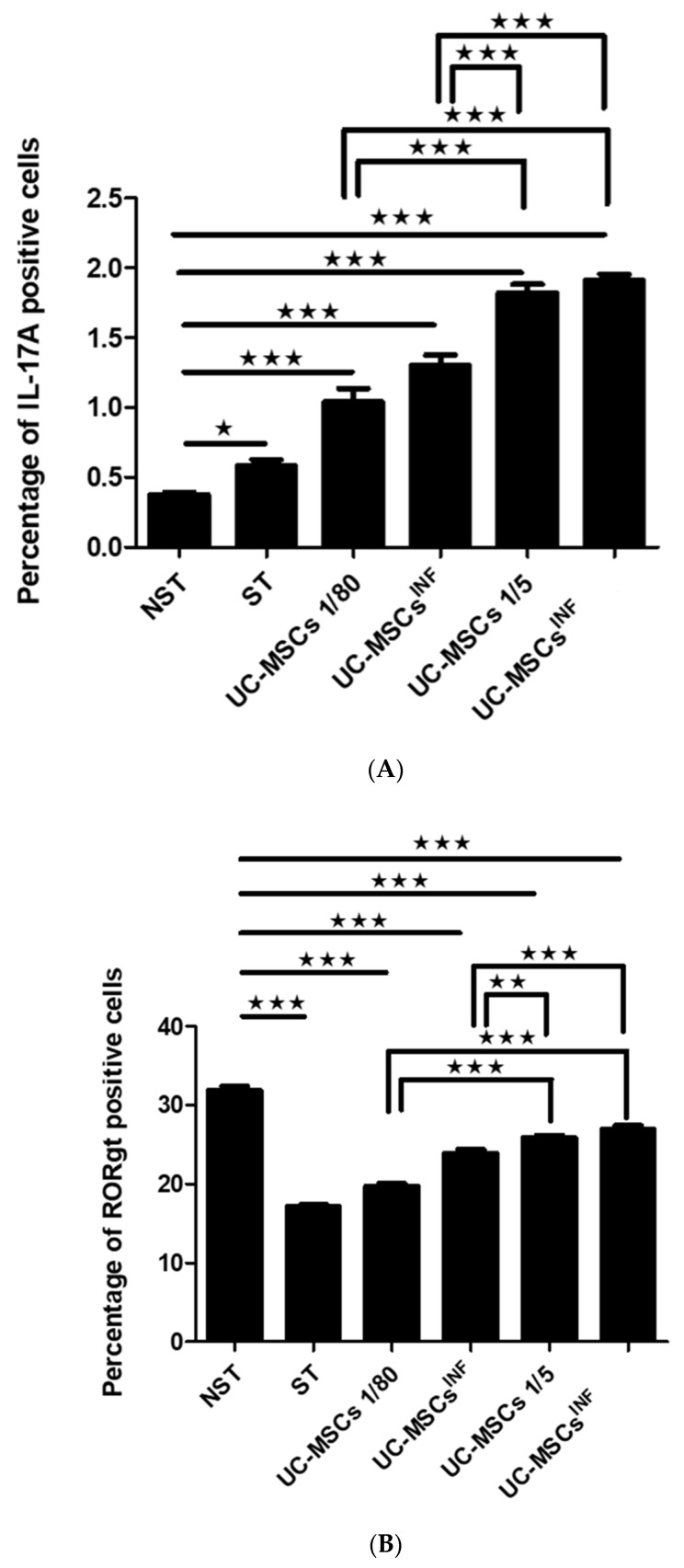

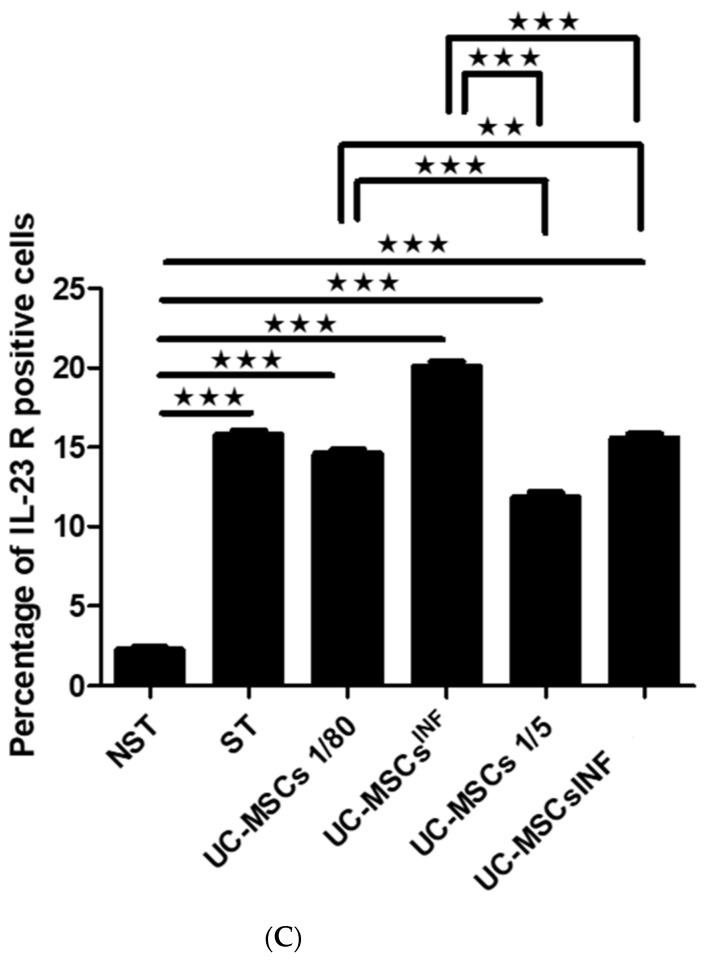
(**A**) The impact of UC-MSCs on IL-17A expression. In normal and inflammatory setting, UC-MSCs (*n* = 5) were cocultured at a 1:5 and 1:80 cell ratio with either unstimulated or PHA/IL-2-stimulated T cells, as indicated. Flow cytometry was used to assess expression levels. The results are given as a percentage ± SEM of positive cells. * *p* ≤ 0.05, *** *p* ≤ 0.001 compared to control. UC-MSCs: umbilical cord mesenchymal stromal/stem cells; UC-MSCs^INF^: preconditioned umbilical cord mesenchymal stromal/stem cells; NST: unstimulated T cells; ST: PHA/IL-2-stimulated T cells. (**B**) The impact of UC-MSCs on RORγt expression. In normal and inflammatory setting, UC-MSCs (*n* = 5) were cocultured at a 1:5 and 1:80 cell ratio with either unstimulated or PHA/IL-2-stimulated T cells, as indicated. Flow cytometry was used to assess expression levels. The results are given as a percentage ± SEM of positive cells. ** *p* ≤ 0.01, *** *p* ≤ 0.001 compared to control. UC-MSCs: umbilical cord mesenchymal stromal/stem cells; UC-MSCs^INF^: preconditioned umbilical cord mesenchymal stromal/stem cells; NST: unstimulated T cells; ST: PHA/IL-2-stimulated T cells. (**C**) The impact of UC-MSCs on IL-23 receptor expression. In normal and inflammatory setting, UC-MSCs (*n* = 5) were cocultured at a 1:5 and 1:80 cell ratio with either unstimulated or PHA/IL-2-stimulated T cells, as indicated. Flow cytometry was used to assess expression levels. The results are given as a percentage ± SEM of positive cells. ** *p* ≤ 0.01, *** *p* ≤ 0.001 compared to control. UC-MSCs: umbilical cord mesenchymal stromal/stem cells; UC-MSCs^INF^: preconditioned umbilical cord mesenchymal stromal/stem cells; NST: unstimulated T cells; ST: PHA/IL-2-stimulated T cells.

Based on the results of flow cytometry analysis shown in Figure 4A, the percentage of T cells expressing IL-17A increased after PHA/IL-2 stimulation (0.58 ± 0.11%) compared to unstimulated T cells alone (0.37 ± 0.05%).

During the coculture process, this proportion significantly increased in cocultures at both cell ratios and both states of cocultured UC-MSCs; nonpreconditioned UC-MSCs cocultured with PHA/IL-2-stimulated T cells at a 1:80 ratio (1.04 ± 0.23%) and at a 1:5 ratio (1.82 ± 0.15%), while preconditioned UC-MSCs cocultured with PHA/IL-2-stimulated T cells at a 1:80 ratio (1.31 ± 0.18%) and at a 1:5 ratio (1.91 ± 0.11%) compared to stimulated T cells (0.58 ± 0.11%) and unstimulated T cells alone (0.37 ± 0.05%).

The preconditioning of UC-MSCs in the coculture also impacted the IL-17A-positive proportion at a ratio of 1:80, as it increased when PHA/IL-2-stimulated T cells were cocultured with preconditioned UC-MSCs (1.31 ± 0.18%) compared to the coculture of nonpreconditioned UC-MSCs with PHA/IL-2-stimulated T cells (1.04 ± 0.23%).

Furthermore, the proportion of IL-17A-positive cells was also affected by the ratio of T cells to UC-MSCs in the coculture, with the 1:5 ratio (1.82 ± 0.15%; 1.91 ± 0.11%) showing a significant increase compared to T cell/UC-MSCs cocultured at a 1:80 ratio (1.04 ± 0.23%; 1.31 ± 0.18%).

### 3.3. Expression of RORγt in T Cells during Coculture with UC-MSCs

Figure 4B shows that in PHA/IL-2-stimulated T cells, the percentage of RORγt-positive T cells (17.18 ± 0.56%) was significantly reduced compared to unstimulated T cells (31.84 ± 1.34%).

Interestingly, T cell/UC-MSC cocultures at both 1:80 and 1:5 ratios, either nonpreconditioned (19.74 ± 0.85%; 25.83 ± 0.78%) or preconditioned (23.89 ± 1.12%; 26.95 ± 1.15%), exhibited a higher RORγt percentage than PHA/IL-2-stimulated T cells (17.19 ± 0.56%) and a lower RORγt percentage than unstimulated T cells alone (31.84 ± 1.34%).

The effect of preconditioning of UC-MSCs was pronounced at the coculture of 1:80, as the percentage of RORγt-positive T cells increased when PHA/IL-2-stimulated T cells were cocultured with preconditioned UC-MSCs (23.89 ± 1.12%) compared to the coculture of nonpreconditioned UC-MSCs with PHA/IL-2-stimulated T cells (19.74 ± 0.84%). The ratio of T cells to UC-MSCs in the coculture also had an influence on the proportion of IL-17A-positive cells, with the 1:5 ratio (25.83 ± 26.95%; 0.77 ± 1.15%) exhibiting a substantial increase compared to T cell/UC-MSC cocultured at a 1:80 ratio (19.74 ± 23.89%; 0.84 ± 1.12%) (see Figure 4B).

### 3.4. Expression of the IL-23 Receptor in T Cells during Coculture with UC-MSCs

The analysis of flow cytometry, depicted in Figure 4C, indicated that when T cells were stimulated with PHA/IL-2, they had much higher levels of IL-23R expression (15.79 ± 0.59%) than when unstimulated (2.24 ± 0.43%).

When T cells were cocultured with UC-MSCs that were either preconditioned or not preconditioned, their levels of IL-23R expression were notably higher (20.09 ± 0.71%; 15.57± 0.69%; 14.59± 0.68%; 11.85 ± 0.75%), respectively, compared to unstimulated T cells alone (2.245 ± 0.43%) (Figure 4C). Compared to stimulated T cells (15.79 ± 0.59%), nonpreconditioned UC-MSCs showed lower levels of IL-23R expression, while preconditioned UC-MSCs showed a higher level of IL-23R expression at a 1:80 ratio. The impact of preconditioning on UC-MSCs was significant during the 1:5 coculture, as the percentage of IL-23R-positive T cells increased when PHA/IL-2-stimulated T cells were cocultured with preconditioned UC-MSCs (15.57 ± 0.69%) compared to the coculture with nonpreconditioned UC-MSCs (11.85 ± 0.75%). The expression levels of IL-23R were also influenced by the ratio of the cells in the coculture. The 1:5 ratio had lower levels of IL-23R than the 1:80 ratio of either preconditioned or nonpreconditioned UC-MSCs (15.57 ± 0.69%; 11.85 ± 0.75%; 20.09 ± 0.71%; 14.59 ± 0.68%, respectively).

### 3.5. Th-17-Associated Cytokine Profile

**Figure 5:**
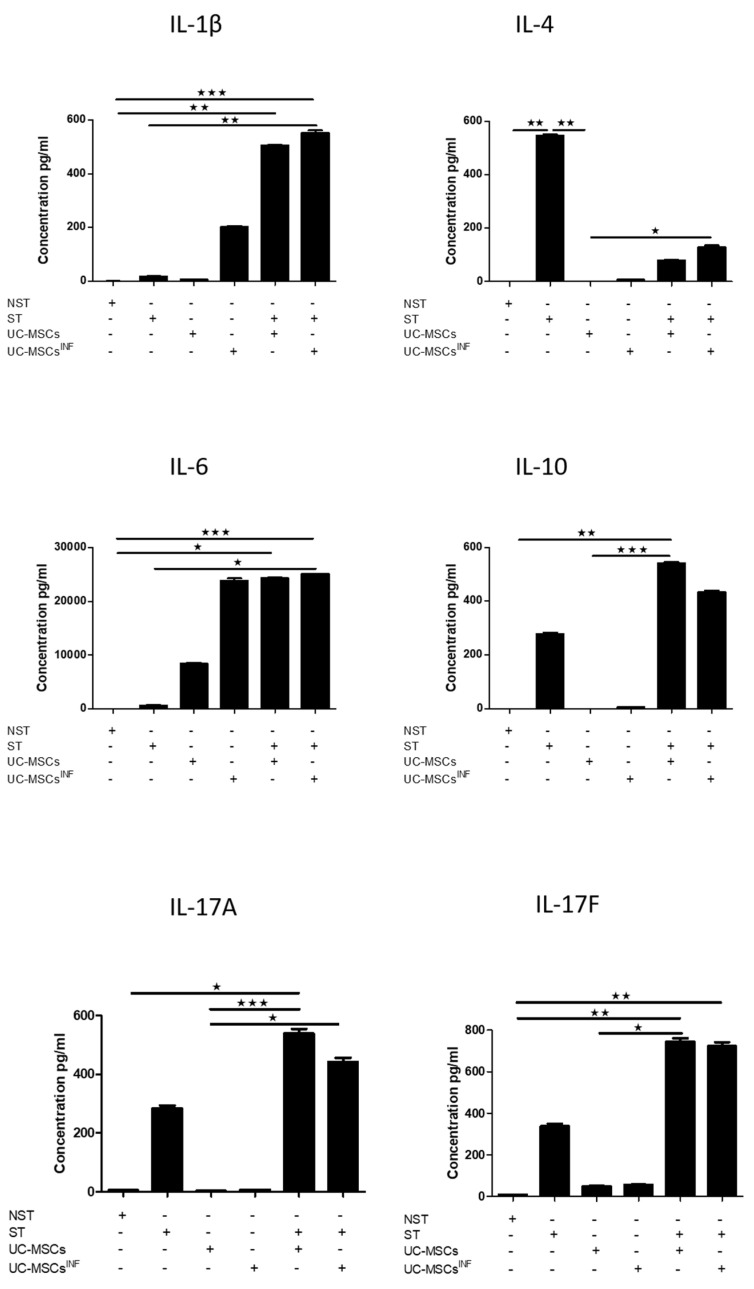
Array profile of IL-1β, IL-4, IL-6, IL-10, IL-17A, and IL-17F cytokines. In normal and inflammatory setting, UC-MSCs (*n* = 5) were cocultured at a 1:5 cell ratio with either unstimulated or PHA/IL-2-stimulated T cells, as indicated. The levels of the various cytokines were determined as previously described in the Materials and Methods section. The data for IL-1β, IL-4, IL-6, IL-10, IL-17A, and IL-17F are shown as concentration ± SEM (pg/mL). * *p* ≤ 0.05, ** *p* ≤ 0.01, and *** *p* ≤ 0.001 compared to the matching control. UC-MSCs: umbilical cord mesenchymal stromal/stem cells; UC-MSCs^INF^: preconditioned umbilical cord mesenchymal stromal/stem cells; NST: unstimulated T cells; ST: PHA/IL-2-stimulated T cells.

**Figure 6:**
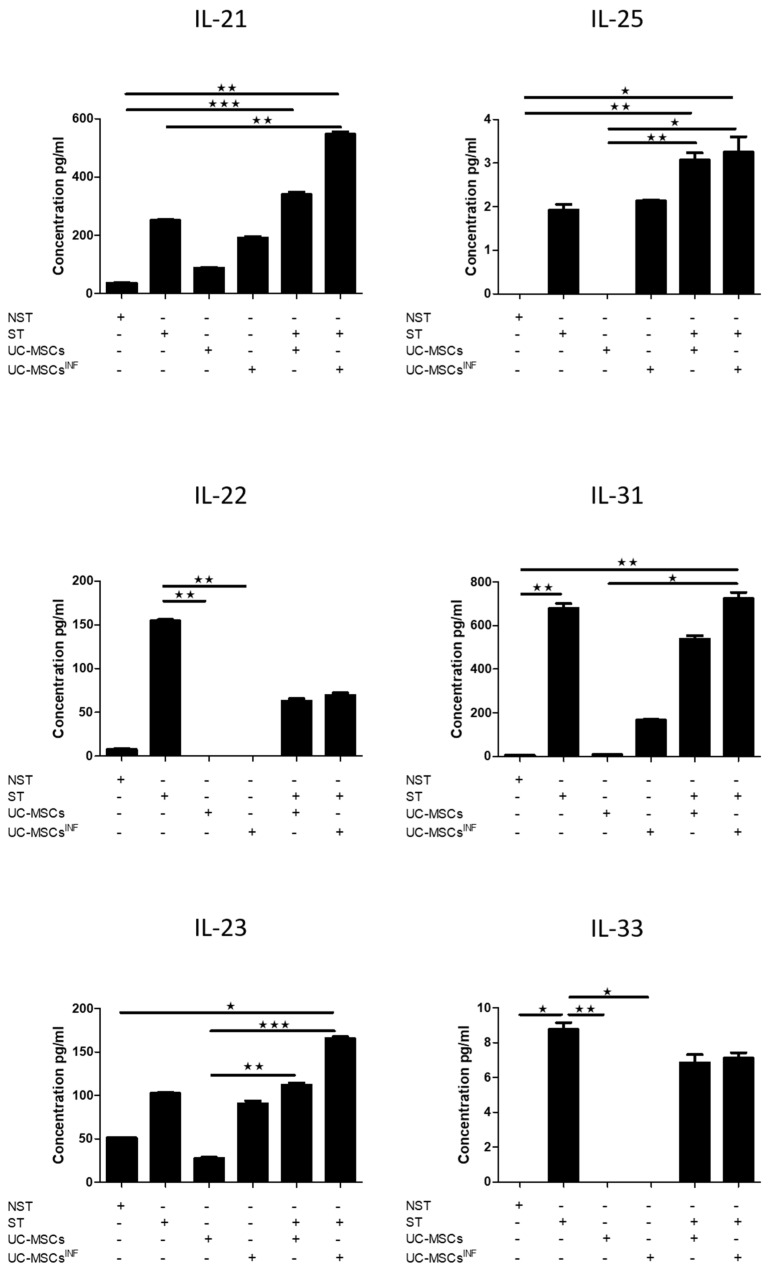
Array profile of IL-21, IL-22, IL-23, IL-25, IL-31, and IL-33 cytokines. In normal and inflammatory setting, UC-MSCs (*n* = 5) were cocultured at a 1:5 cell ratio with either unstimulated or PHA/IL-2-stimulated T cells, as indicated. The levels of the various cytokines were determined as previously described in the Materials and Methods section. The data for IL-21, IL-22, IL-23, IL-25, IL-31, and IL-33 are shown as concentration ± SEM (pg/mL). * *p* ≤ 0.05, ** *p* ≤ 0.01, and *** *p* ≤ 0.001 compared to the matching control. UC-MSCs: umbilical cord mesenchymal stromal/stem cells; UC-MSCs^INF^: preconditioned umbilical cord mesenchymal stromal/stem cells; NST: unstimulated T cells; ST: PHA/IL-2-stimulated T cells.

**Figure 7:**
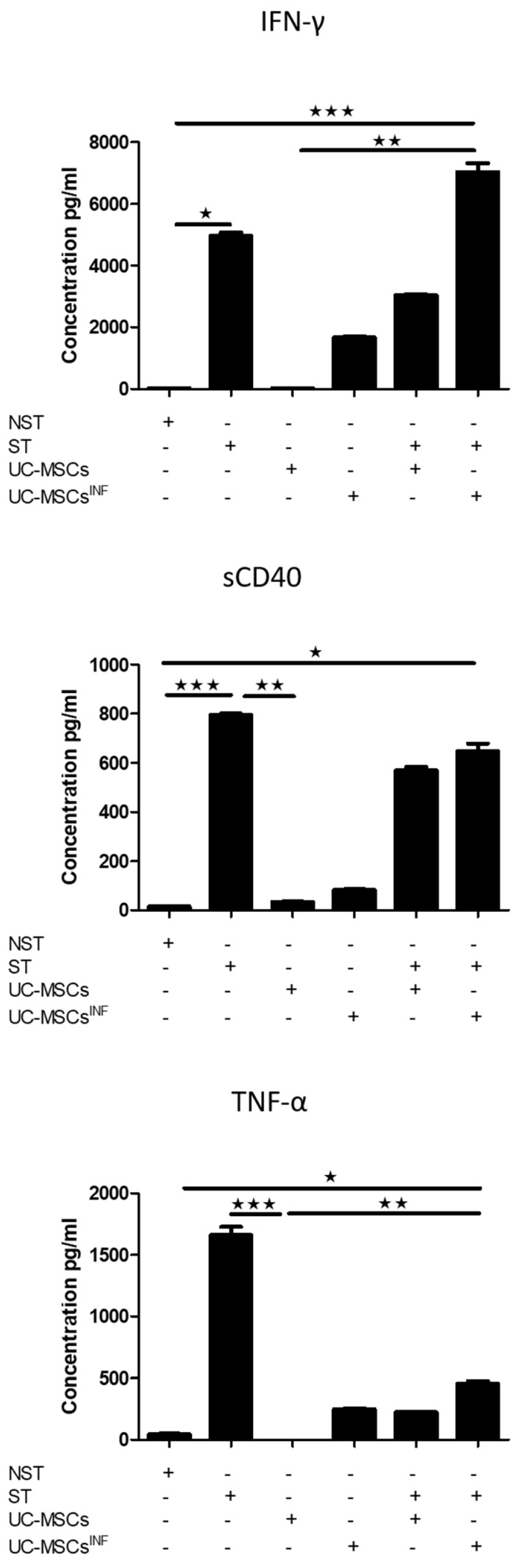
Array profile of INF-γ, sCD40, and TNF-α cytokines. In normal and inflammatory setting, UC-MSCs (*n* = 5) were cocultured at a 1:5 cell ratio with either unstimulated or PHA/IL-2-stimulated T cells, as indicated. The levels of the various cytokines were determined as previously described in the Materials and Methods section. The data for INF-γ, sCD40, and TNF-α are shown as concentration ± SEM (pg/mL). * *p* ≤ 0.05, ** *p* ≤ 0.01, and *** *p* ≤ 0.001 compared to the matching control. UC-MSCs: umbilical cord mesenchymal stromal/stem cells; UC-MSCs^INF^: preconditioned umbilical cord mesenchymal stromal/stem cells; NST: unstimulated T cells; ST: PHA/IL-2-stimulated T cells.

The cytokine array profile allows the detection and measurement of fifteen Th-17 biomarkers as presented in Figure 5, Figure 6 and Figure 7 for the 1/5 cell ratio and as Supplementary Figure S1 for the 1/80 cell ratio. Based on the results, PHA/IL-2-stimulated T cells were compared to unstimulated T cells, which produced significantly higher levels of IL-4 (545.78 ± 7.59 pg/mL), IL-10 (277.83 ± 9.45 pg/mL), IL-17A (283.41 ± 16.81 pg/mL), IL-17F (337.93 ± 21.76 pg/mL), IL-21 (251.41 ± 7.06 pg/mL), IL-22 (154.62 ± 2.59 pg/mL), IL-23 (102.39 ± 1.68 pg/mL), IL-25 (1.92 ± 0.23 pg/mL), IL-31 (678.71 ± 39.14 pg/mL), IL-33 (8.75 ± 0.68 pg/mL), IFN-γ (4958.21 ± 191.23 pg/mL), sCD40 (796.49 ± 8.29 pg/mL), and TNF-α (1662.91 ± 110.70 pg/mL). PHA/IL-2-stimulated T cells also produced measurable levels of IL-1β (17.18 ± 1.71 pg/mL) and IL-6 (648.78 ± 11.96 pg/mL), whereas unstimulated T cells generated only minimal quantities.

UC-MSCs that underwent preconditioning had elevated levels of specific cytokines, such as IL-1β (109.86 ± 5.08 pg/mL; 201.70 ± 7.14 pg/mL), IL-6 (24,216.54 ± 1749.32 pg/mL; 23,841.41 ± 777.19 pg/mL), IL-21 (183.83 ± 11.33 pg/mL;191.27 ± 8.48 pg/mL), IL-23 (69.45 ± 2.96 pg/mL; 90.67 ± 5.14 pg/mL), IL-25 (1.73 ± 0.43 pg/mL; 2.13 ± 0.05 pg/mL), IL-31 (266.50 ± 18.59 pg/mL; 167.48 ± 5.29 pg/mL), IFN-γ (1893.32 ± 125.66 pg/mL; 1672.65 ± 60.97 pg/mL), sCD40 (82.52 ± 5.85 pg/mL; 82.85 ± 5.21 pg/mL), and TNF-α (289.87 ± 20.62 pg/mL; 249.39 ± 12.18 pg/mL), in comparison to nonpreconditioned UC-MSCs. Furthermore, they also generated slightly more IL-4 (6.36 ± 0.77 pg/mL; 6.55 ± 0.85 pg/mL), IL-10 (10.06 ± 1.50 pg/mL; 5.88 ± 1.05 pg/mL), Il-17A (5.18 ± 0.75 pg/mL; 6.15 ± 0.63 pg/mL), and IL-17F (60.73 ± 4.11 pg/mL; 56.41 ± 1.55 pg/mL). However, neither cell line produced detectable levels of IL-22 (0.00 pg/mL; 0.00 pg/mL) or IL-33 (0.00 pg/mL; 0.00 pg/mL).

Cocultures of PHA/IL-2-stimulated T cells with preconditioned UC-MSCs at both 1:80 and 1:5 ratios significantly stimulated the production of the following cytokines: IL-1β (228.06 ± 9.68 pg/mL; 551.94 ± 16.43 pg/mL), IL-6 (24,648.88 ± 728.82 pg/mL; 25,065.74 ± 27.02 pg/mL), IL-10 (365.62 ± 9.53 pg/mL; 433.17 ± 10.60 pg/mL), IL-17F (801.71 ± 46.63 pg/mL;722.20 ± 34.81 pg/mL), IL-21 (482.18 ± 6.73 pg/mL; 546.63 ± 15.10 pg/mL), IL-23 (153.36 ± 4.74 pg/mL; 165.28 ± 4.02 pg/mL), IL-25 (3.22 ± 0.48 pg/mL; 3.25 ± 0.61 pg/mL), and γ-γ (6220.69 ± 359.57 pg/mL; 7028.29 ± 501.03 pg/mL) compared to PHA/IL-2-stimulated T cells alone. However, the levels of IL-31 (767.01 ± 16.54 pg/mL; 724.06 ± 49.16 pg/mL) and IL-33 (8.18 ± 0.50 pg/mL; 7.11 ± 0.55 pg/mL) remained unchanged; the expression of IL-4 (168.74 ± 7.81 pg/mL; 128.90 ± 11.12 pg/mL), IL-22 (83.57 ± 5.59 pg/mL; 69.54 ± 4.91 pg/mL), and sCD40 decreased (645.87 ± 42.79 pg/mL; 648.54 ± 51.86 pg/mL); and the levels of TNF-α increased at a 1:80 ratio (2306.91 ± 210.22 pg/mL) and decreased at a 1:5 ratio (455.94 ± 37.22 pg/mL). IL-17A only increased at a 1:5 ratio (5.18 ± 0.75 pg/mL; 6.15 ± 0.63 pg/mL). When nonpreconditioned UC-MSCs were cocultured with PHA/IL-2-stimulated T cells, at both ratios, there was an increase in the production of IL-1β (123.37 ± 3.94 pg/mL; 505.97 ± 4.75 pg/mL), IL-6 (23,949.59 ± 595.70 pg/mL; 24,318.82 ± 276.64 pg/mL), IL-10 (488.07 ± 11.31 pg/mL; 541.49 ± 7.50 pg/mL), IL-17A (515.70 ± 6.86 pg/mL; 538.23 ± 31.60 pg/mL), 17F (778.04 ± 36.41 pg/mL; 744.02 ± 34.36 pg/mL), IL-21 (510.19 ± 6.98 pg/mL;339.57 ± 18.54 pg/mL), and IL-25 (3.44 ± 0.47 pg/mL; 3.07 ± 0.34 pg/mL). On the other hand, IL-4 (220.39 ± 3.11 pg/mL; 79.06 ± 5.40 pg/mL), IL-22 (84.53 ± 9.96 pg/mL; 62.86 ± 6.01 pg/mL), IFN-γ (4088.09 ± 44.82 pg/mL; 3034.08 ± 68.24 pg/mL), sCD40 (581.03 ± 59.47 pg/mL; 570.43 ± 28.37 pg/mL), and TNF-α (1415.87 ± 36.27 pg/mL; 224.83 ± 10.22 pg/mL) were decreased. IL-23 (130.10 ± 3.14 pg/mL; 112.03 ± 4.61 pg/mL) was only increased at a ratio of 1:80, and IL-31 (708.17 ± 38.65 pg/mL; 537.60 ± 31.65 pg/mL) was decreased at a ratio of 1:5, while IL-33 (7.45 ± 1.19 pg/mL; 6.86 ± 0.91 pg/mL) levels remained unchanged compared to PHA/IL-2-stimulated T cells alone. Cocultures of PHA/IL-2-stimulated T cells with UC-MSCs, whether preconditioned or nonpreconditioned, revealed increased levels of all the previously stated cytokines compared to unstimulated T cells.

When comparing the coculture of PHA/IL-2 stimulated T cells with preconditioned UC-MSCs to the coculture of PHA/IL-2 stimulated T cells with nonpreconditioned UC-MSCs at both 1:80 and 1:5 cell ratios, respectively, it was observed that the former had higher levels of Il-1β (228.06 ± 9.68 pg/mL; 551.94 ± 16.43 pg/mL), Il-23 (153.36 ± 4.74 pg/mL; 165.28 ± 4.02 pg/mL), IFN-γ (6220.69 ± 359.57 pg/mL; 7028.29 ± 501.03 pg/mL), and TNF-α (2306.91 ± 210.22 pg/mL; 455.94 ± 37.22 pg/mL), while having lower levels of Il-10 (365.62 ± 9.53 pg/mL; 433.17 ± 10.60 pg/mL), IL-17A (5.18 ± 0.75 pg/mL; 6.15 ± 0.63 pg/mL), and Il-22 (83.57 ± 5.59 pg/mL; 69.54 ± 4.91 pg/mL). The levels of IL-6 (24,216.54 ± 1749.32 pg/mL; 25,065.74 ± 27.02 pg/mL), IL-17F (801.71 ± 46.63 pg/mL; 722.20 ± 34.81 pg/mL), IL-25 (3.22 ± 0.48 pg/mL; 3.25 ± 0.61 pg/mL), IL-33 (8.18 ± 0.50 pg/mL; 7.11 ± 0.55 pg/mL), and sCD40 (645.87 ± 42.79 pg/mL; 648.54 ± 51.86 pg/mL) were similar in both groups, while at 1:5, preconditioned UC-MSCs had higher levels of IL-21 (482.18 ± 6.73 pg/mL; 546.63 ± 15.10 pg/mL) and IL-31 (767.01 ± 16.54 pg/mL; 724.06 ± 49.16 pg/mL), and at the 1:80 ratio, preconditioned UC-MSCs had lower levels of IL-4 (168.74 ± 7.81 pg/mL; 128.90 ± 11.12 pg/mL) but higher levels of this cytokine at a 1:5 ratio.

## 4. Discussion

The use of stem cells, including MSCs, in tissue regeneration and wound healing holds a great challenge for modern care strategies. UC-MSCs are a valuable type of MSC with unique therapeutic features representing interesting alternative sources compared to classical sources [19]. They can be easily obtained from the umbilical cord without any invasive procedures or ethical concerns. During injury, MSCs, through their immunomodulatory properties, establish a healing and immune-tolerant environment suitable for tissue repair [20]. Mechanistically, MSCs demonstrate plasticity in their fate by acting as sensors and regulators following tissue damage or inflammatory events. To exert their effects, MSCs release trophic and regulatory factors required for controlling the immune response and maintaining tissue homeostasis [21]. In a previous study, the morphology, proliferation and phenotype of UC-MSCs were not modified by inflammatory treatment. T cell proliferation was significantly decreased in the presence of UC-MSCs in a dose- and inflammatory-dependent manner [22].

Accumulating evidence indicates that IL-17 has important context- and tissue-dependent roles in maintaining an individual’s health status during the response to injury, physiological stress, and infection. Upon tissue damage, inflammation is an early event that is orchestrated by a multitude of innate and adaptive immune cell subsets, including Th-17 cells. These cells are attracted by chemokines, and their associated cytokines can thus impact the healing process. The inflammatory phase is important for tissue regeneration due to the secretion by immune cells of a panel of proinflammatory cytokines and growth factors [23]. Owing to their plasticity, Th-17 cells may transition between pro- and anti-inflammatory states, which may influence the immunological and inflammatory processes linked to wound healing [24]. An important future goal of using UC-MSCs for tissue-reparative purposes is to understand their interplay and regulation of the Th-17 pathway during tissue injury and inflammation. In this study, we investigated Th-17-specific markers, including interleukin-17A, IL-23R, and RORγt, as well as the Th-17-specific pathway secretome in the presence of UC-MSCs. IL-17A, RORγt, and IL-23R play a crucial role in the development, regulation, and function of Th-17 cells.

IL-17A, formerly known as cytotoxic T-lymphocyte antigen (CTLA)-8, is the hallmark cytokine of Th-17 cells [25]. Produced by Th-17 cells, it can induce inflammation and attract immune cells [26]. RORγt is a major transcription factor that regulates IL-17A production and Th-17 cell development [27]. IL-23 binds to the receptor IL-23R, which enables the expansion and activation of Th-17 cells [28]. Thus, IL-17A, RORγt, and IL-23R play a crucial role in the development, regulation, and function of Th-17 cells. When UC-MSCs and PHA/IL-2-stimulated T cells were cocultured, an increase in IL-17A and RORγt expression was observed, while IL-23R expression was differentially regulated. These changes depended on the cell ratio and the inflammatory preconditioning state of UC-MSCs. We have previously shown that MSCs derived from foreskin (FSK), bone marrow (BM), and adipose tissue (AT) differentially impact Th-17 markers during coculture with PHA/IL-2-stimulated T cells. Regardless of the T cell/MSC ratio, we observed a significant increase in IL-17A expression associated with an increase in IL-23 receptor expression [16]. In contrast, AT-MSCs induced a dose-dependent increase in IL-17A and ROR-γt expression without influencing IL-23 receptor (IL-23R) expression [29]. Finally, cocultures of either preconditioned or nonpreconditioned BM-MSCs significantly induced increases in IL-17A and RORγt expression [30]. These observations indicate that the impact of MSCs on IL-17A, RORγt, and IL-23R expression by stimulated T cells depends on the source of MSCs, the presence or absence of an inflammatory setting, and the cell ratio of the coculture. Of importance, the cytokine IL-17A can limit the pathogenicity of Th-17 via a negative-feedback loop induced by the autocrine production of IL-24 [31].

The lineage-defining transcription factor RORγt in Th-17 cells is essential and sufficient to induce Th-17 lineage fate. Indeed, several studies revealed that multiple transcriptional regulators contribute to the full Th-17 differentiation program through several mechanisms, including binding to specific regions of IL-17a and Rorc genes, interacting and synergizing with RORγt, or facilitating the recruitment of other proteins on IL-17a or Rorc promoters [32]. RORα functions as a key regulator of the Th-17 effector program through direct regulation of sustained RORγt expression during chronic inflammation. RORα enforces the stability of the Th-17 cell effector program by binding to a Rorc cis-regulatory element [33]. Coexpression of RORα4 and RORγt causes a synergistic increase in IL-17A, which together regulates Th-17 cell differentiation but is not sufficient to generate and specify the full Th-17 program. Of importance, the expression of RORγ is reported to be unstable and influenced by environmental cues, allowing for Th-17 heterogeneity and plasticity [34].

Here, we also investigated the impact of UC-MSCs on Th-17 cells by studying the regulation of the secretome (IL-1β, IL-4, IL-6, IL-10, IL-17A, IL-17F, IL-22, IL-21, IL-23, IL-25, IL-31, IL-33, INF-γ, sCD40, and TNF-α) involved in the Th-17 immune response pathway. Both the cell ratio and inflammatory setting used for the culture of UC-MSCs induced substantial changes in the secretory profile of these cytokines. Such modulation of the secretion profile occurs independently of the source of MSCs, as previously published [16,29,30].

Interleukin-17 (IL-17), also known as IL-17A, is the main member of the IL-17 family, which includes six members: IL-17A, IL-17B, IL-17C, IL-17D, IL-17E (also named IL-25), and IL-17F. In addition to IL-17A, Th-17 cells produce IL-17F, IL-21, and IL-22, inducing the recruitment of neutrophils and macrophages to tissues, which collectively ensure an appropriate defense against pathogens [35]. In particular, the binding of IL-17A with its receptor activates target cells, such as epithelial cells, endothelial cells, and fibroblasts, and induces CXCL1, CXCL2, and CXCL8, which attract myeloid cells, such as neutrophils, to infected or injured tissue, thus contributing to microbial clearance [36]. In the present study, the increase in the levels of IL-17A and IL-17F during coculture of stimulated T cells and UC-MSCs clearly depended on both the cell ratio and the inflammatory preconditioning state. It is important to note that tissue inflammation could occur due to the production of IL-17F and IL-17A without the involvement of IL-23 [37]. These cytokines interact with their unique receptors and activate various cell types [38,39]. The Th-17 cytokine pathway may support cartilage degradation and bone resorption during rheumatoid arthritis (RA), intensifying the chronic inflammatory process. The blockade of some cytokines using monoclonal antibodies might be a new therapeutic approach to reduce inflammation and enhance cartilage repair mechanisms in patients with RA [40].

Through interleukin (IL)-6, TGF-β, and IL-1β signaling, Th-17 cells might be induced by naïve T cells. Once expanded, they secrete cytokines, such as tumor necrosis factor (TNF)-α, IL-17, and IL-22, which may promote cell survival and proliferation and thus impact tissue regeneration [41].

Our findings indicate that IL-21 is substantially increased during coculture of stimulated T cells and UC-MSCs in a cell ratio and inflammatory preconditioning manner. It has been demonstrated that IL-21 controls the generation of Th-17 cells in vitro and in vivo. IL-6 induced IL-21 production in a STAT3-dependent and RORγ-independent manner. IL-21 in combination with TGF-β induces the expression of IL-23R and RORγ, leading to IL-17 production from naïve CD4 cells [42].

Interleukin-22 (IL-22), a member of the IL-10 cytokine family, participates in the communication between the immune system and peripheral tissues. Regardless of the preconditioning status of MSCs, coculturing T cells with UC-MSCs resulted in a general increase in several Th-17-cell-derived cytokines in a cell-ratio-dependent manner. IL-22 is known to play a key role in tissue regeneration and wound healing by activating genes linked to proliferation and cell survival [43]. IL-1β can act both independently of and synergistically with IL-23 to induce IL-22 [44]. It was reported that cocultures of UC-MSCs and PBMCs demonstrate possible regulation of the relative content and function of Th-17 lymphocytes in different inflammatory and immunological models [45].

In our setting, UC-MSCs are likely to increase the expression of IL-23R on activated T cells depending on the cell ratio and inflammation signal. IL-23 signaling, which is not required for the initial differentiation, is crucial for the final differentiation, expansion, and maintenance of Th-17 cells. This is likely because naïve T cells do not express IL-23R. As the induction of IL-23R expression depends on factors that initiate Th-17 differentiation (IL-6 and IL-21), IL-23 may synergize with IL-6 to induce Th-17 differentiation [46]. IL-23 selectively expands a unique population of TH cells that selectively express IL-17 and IL-17F [47]. We showed that the level of IL-6 was substantially increased in UC-MSC and activated T cell cocultures. Since IL-6 signaling can induce the expression of Rorc, IL-17, and IL-23R, which are required in the early priming phase of Th-17 cells [48], it is possible that UC-MSCs promote a Th-17-cell-like profile.

Depending on the context, IL-6 can have both proinflammatory and anti-inflammatory effects [49]. IL-6 can trigger the production of IL-17 cytokines in a STAT3-dependent manner [50], and persistent IL-6 signaling is also needed to sustain the Th-17 phenotype [51]. Several studies have shown that IL-6 promotes the differentiation of Th-17 cells from naïve cells along with TGF-β [52] and that TNF-α and IL-1β can further increase IL-17 expression [53]. TGF-β and IL-6 support the differentiation of IL-17-producing T cells from naïve cells, and tumor necrosis factor alpha (TNF-α) and IL-1β further amplify IL-17 expression. IL-1β was found to promote Th-17 development/expansion in the presence of IL-6 and TGF-β. Moreover, IL-23 cooperates with IL-1α or IL-1β and enhances IL-17 production, independent of T cell receptor stimulation. It was reported that IL-6 is capable of inhibiting TGF-β-dependent Foxp3+ Treg cell induction. These studies suggest that there is not only functional antagonism between Th-17 and Treg cells but also a dichotomy in their generation that depends on whether they are activated in the presence of proinflammatory conditions [54]. Accordingly, the balance between TGF-β and proinflammatory cytokines such as IL-6 might influence the final outcome in the differentiation process from naïve T helper cells to different effector subsets.

Various cytokines, via a complex transcriptional network, are involved in the differentiation of Th-17 cells. Understanding the whole picture could facilitate the design of new therapeutic strategies targeting Th-17 cells in the presence of UC-MSCs. In our study, IL-1β was mainly derived from UC-MSCs and was significantly increased upon inflammatory preconditioning. IL-1β is an important cytokine for the differentiation of Th-17 cells with multiple signaling effects, including RORγt expression [55]. IL-1β is a proinflammatory cytokine that plays a significant role in the immune response and inflammatory processes [56]. IL-1β triggers the production of other cytokines, including IL-6 and IL-23 [57]. IL-1β can suppress the inhibitory effects of IL-2 on IL-17 production through induction of IL-1R, IL-23R, and RORγt.

The secretion profile of TNF-α by stimulated T cells during coculture with UC-MSCs is also influenced by the cell ratio and the inflammatory setting. TNF-α, an important proinflammatory cytokine, plays a key role during inflammation and subsequent wound healing. TNF-α signaling mediates the secretion of inflammatory factors; regulates cell survival, proliferation, and death; and influences epithelial wound healing [58]. It also stimulates the production of IL-6, IL-1β, and other proinflammatory mediators and may therefore influence the development of Th-17 cells [59]. It appears that TNF-α may influence how IL-23R is expressed, with TNFR2+ T cells showing significantly higher levels of IL-23R, IFN-γ, and IL-17A than TNFR2- cells [60]. The effects of TNF-α depend on the dose and duration of exposure. Specifically, low levels of TNF-α promote inflammation and stimulate the production of macrophage-derived growth factors, facilitating wound healing. However, long exposure to high levels of TNF-α may alter healing by leading to reduced production of ECM components while promoting the expression of metalloproteinases (MMP-1, MMP-2, MMP-3, MMP-9, MMP-13, and MT1-MMP). In a parallel study, cocultures of cord blood (CB) and Wharton’s jelly (WJ) with PBMCs increased the intracellular levels of IFN-γ, IL-17, and IL-4 and interestingly decreased their supernatant levels [61]. Accordingly, these results indicate that the observed differential immunologic effects during coculture of MSCs and lymphocytes depend on the incubation time and ratios.

Interestingly, the levels of IFN-γ were significantly increased during the coculture of PHA/IL-2-stimulated T cells, while those of IL-4 were decreased, suggesting a modulation of Th-17 plasticity by UC-MSCs depending on their cellular and cytokine environment. Th-17 cells have been reported to produce IFN-γ and IL-4, which are considered Th1 and Th2 signature cytokines, respectively. IFN-γ, IL-12, or IL-4, which are important for TH1 and TH2 differentiation, have been shown to be dispensable for Th-17 cell differentiation in vitro and in vivo. IL-4 is a crucial Th2 cytokine; it plays a role in T cell immunity and has also been associated with tissue repair and homeostasis [62]. Th-17 differentiation necessitates the transcription factor STAT3, which inhibits IL-4 synthesis [63]. Even IFN-γ and IL-4 are known to negatively regulate Th-17 differentiation, and other studies have reported the presence of IL-17-producing CD4 T cells that coexpress IFN-γ. This suggests that IFN-γ contributes to the pathogenic function of Th-17 cells. Studies have identified a subset of IL-17/IFN-γ double-positive T cells, namely, Th-17/Th1 cells, in inflamed tissues or blood. Th-17 cells have been found to exhibit high plasticity because they convert to Th-17/Th1 cells in inflammatory environments [64]. These observations raise the possibility of an interconversion between different T helper subsets and suggest a higher plasticity for Th-17 cells. It is known that in the absence of TGF-β, IL-1β-induced Th-17 cells are T-bet positive and coexpress IFN-γ, the signature cytokine of TH1 cells. Human Th-17 cells comprise heterogeneous subsets including IFN- γ–producing cells with distinct properties from the Th1 lineage. In addition to IL-17 single-producing T cells, IL-17/IFN-γ double-positive T cells are found in significantly elevated numbers in inflamed tissues. Such a profile suggests some relationship between Th-17 and Th1 differentiation programs. However, little is known about the relative profile and function of these cytokine-producing subsets, and assessing the heterogeneity within each population is important for deciphering their origin and respective roles during inflammation and host defense [65].

However, Th-17 cells are also potentially pathogenic and can result in chronic inflammatory conditions or carcinogenesis if not tightly controlled. Therefore, the immune system needs control mechanisms to keep Th-17 cells in check and limit the regenerative program, and several mechanisms to control Th-17 cells have been reported. Thus, Th-17 cells may convert from a proinflammatory phenotype to an anti-inflammatory one by changing their cytokine panel. By acquiring IL-10, it limits their own pathological potential. When stimulated T cells were cocultured with either preconditioned or nonpreconditioned UC-MSCs at both cell ratios, the levels of IL-10 significantly increased. It has been demonstrated that these regulatory Th-17 cells can suppress other effector T cells in vitro and display a noninflammatory gene expression profile [65]. When the IL-10 receptor is expressed on Th-17 cells, it significantly reduces the expression of IL-17A and IL-1β. However, it did not affect RORγt expression, indicating that this receptor may participate in the regulation of Th-17 cell function, not polarization [66].

In addition, we observed that IL-25 is substantially increased during coculture of stimulated T cells and UC-MSCs in a cell ratio and inflammatory preconditioning manner. IL-25, also known as IL-17E, is a cytokine belonging to the IL-17 family. IL-25 is abundantly expressed by Th2 cells and various kinds of epithelial cells. The cellular targets of IL-25 include T cells, myeloid lineage cells, and nonhematopoietic cell populations (e.g., fibroblasts and mesenchymal cells). IL-25 is an alarm signal generated upon cell injury or tissue damage to activate immune cells through interaction with IL-17RA and IL-17RB receptors. The binding of IL-25 to the IL-17RA/IL-17RB complex not only initiates and maintains type 2 immunity but also activates multiple downstream signaling pathways, including NF-κB, MAPK, JAK, and STAT3, which play diverse roles in the self-renewal, survival and apoptosis of cells as well as inflammation [67]. Interestingly, both IL-31 and IL-33 levels remained unchanged. IL-31 controls signaling and regulates a large number of biological functions: it induces proinflammatory cytokines, regulates cell proliferation, and is involved in tissue remodeling. On the other hand, IL-33 has been identified as an “alarmin” released from epithelial cells and from different human tissues and organs after damage following an inflammatory process [68].

Collectively, depending on the inflammatory milieu and the profile of the cytokines produced by T cell subsets, the phenotype and plasticity of human Th-17 cells after coculture with UC-MSCs are modulated and provide new insights into the heterogeneity, functionality, and relationship between human Th-17 cells within their tissue microenvironment. The stimulation by PHA/IL2 increased the percentage of T cells expression of IL-17A and IL-23R, while it decreased that of RORgt. In the presence of UC-MSCs, the expression of IL-17 and RORγt A in stimulated T cells has increased in a cell-ratio-dependent manner regardless of the inflammatory setting. In contrast, the expression of IL-23R decreased in the presence of UC-MSCs in a cell-ratio-dependent manner. Specifically, inflammation setting tends to restore the expression of IL-23R. Regarding the cytokines modulated during these cocultures, we have observed that the expression of IL-1, IL-6, IL-10, IL-17A, IL-17F, IL-21, IL-23, and Il-25 was mainly increased by preconditioned UC-MSCs during high-cell-ratio coculture. In contrast, the expression of IL-4, Il-22, and sCD40 was substantially reduced after coculture with UC-MSCs regardless of the inflammatory setting and cell ratio. Among all the cytokines analyzed, IL-6, IL-17, IL-21, and IL-1 are the main group that have demonstrated significant differential expression profiles, with IL-6 being the most affected cytokine. Indeed, the secretion of IL-6 was substantially increased by inflammation reaching very high levels. It has been shown that the differentiation of distinct T helper cell subsets including Th-17 cells may be linked to distinct signaling modalities of IL-6 [69]. Thus, ongoing classic IL-6 signaling underpins the Th-17 program and is required for Th-17 cell maintenance and function [51]. Based on these flow cytometry and cytokine array analysis, it is likely that UC-MSCs are able to modulate the Th-17 immune response pathway, promoting thus a Th-17-like cell population. There are different types of inflammatory factors in the process of tissue damage, repair, and remodeling, along with disease progression. These factors are involved in boosting of the functions of MSCs by promoting their migration to damaged tissues and shaping their regulatory role according to the local microenvironment conditions [70]. The development of new therapeutic approaches for the management of inflammatory diseases may be based on the modulation of Th-17 cell plasticity.

## 5. Conclusions

UC-MSCs are thus sensors and switchers of inflammation, which is an essential component of tissue healing. The coculture of T cells and UC-MSCs resulted in the generation of a Th-17-like profile, which may be required for tissue repair according to the local microenvironment. Protein network and functional enrichment analysis indicated that several biological process, molecular functions, and cellular components linked to distinct Th-17 signaling interactions are involved in several trophic, inflammatory, and immune network responses. The bidirectional communication between UC-MSCs and Th-17 cells, and the plasticity of both cells, may result in a multifaceted cytokine interplay. Such changes are context-dependent, influenced by the cell ratio and the inflammatory setting. Such plasticity in functions and phenotype allows both cell populations to adapt their fate to tissue challenges and effectively elicit the proper therapeutic effect.

### Perspectives

**Figure 8:**
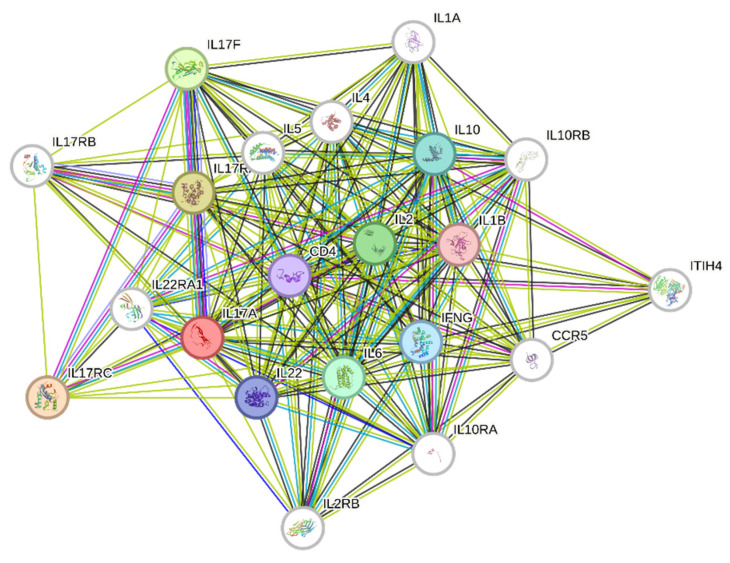

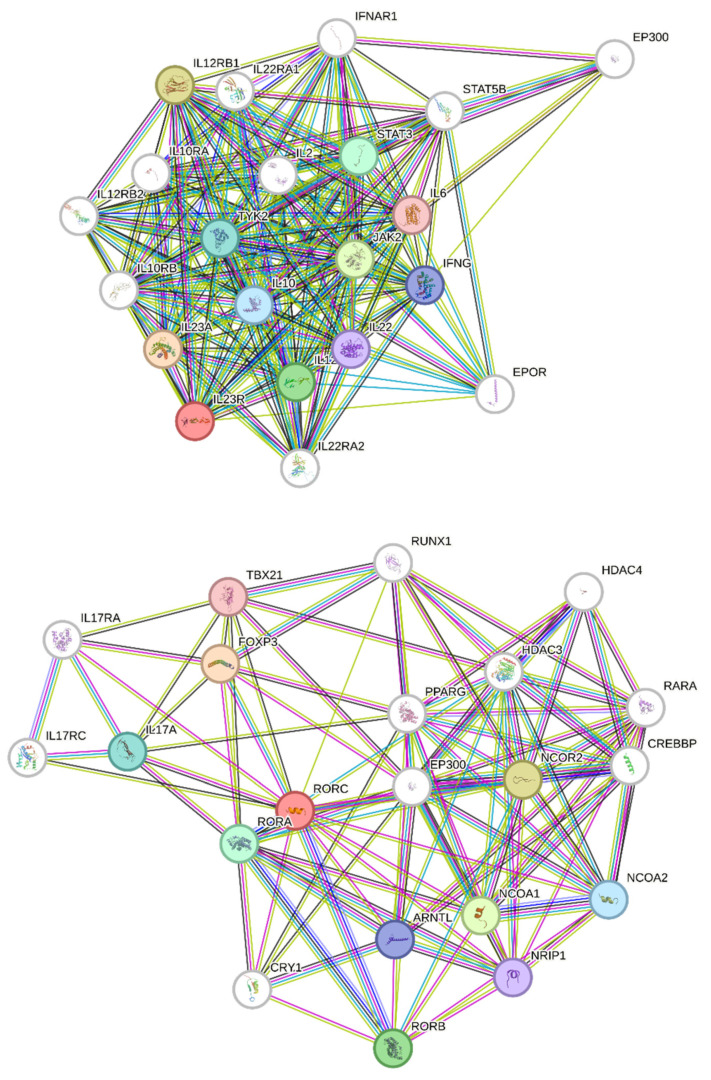
STRING 11.0 analysis of known and predicted IL-17A–, RORγt–, and IL-23R–protein interactions. The interactions include direct (physical) and indirect (functional) associations. Network nodes represent proteins, and edges represent protein-protein associations (specific and meaningful). The lines represent the existence of the several types of evidence used in predicting the associations (high confidence score 0.9). The interactions are shown in different colors: black is coexpression; dark blue is co-occurrence; purple is experimental evidence; light green is text mining.

Depending on the injured tissue context—infected or not, acute or chronic associated inflammation—we suggest that these Th-17-derived cytokines modulated by UC-MSCs could be used to regulate the wound-healing process, modulate stem cell engraftment, and therefore accelerate tissue repair. However, the precarious balance between beneficial and deleterious properties of numerous cytokines, especially those derived from Th-17 cells after modulation by UC-MSCs, depending on the target cells, the inflammatory, and/or the infectious states, should make us more cautious before using cellular therapeutic products in the context of regenerative medicine. Further studies both in vitro (3D injury model) as well as in vivo are needed to evaluate the beneficial/deleterious effects of UC-MSCs on acceleration or slowing down of the healing kinetics in infected wounds, in particular in the presence of proinflammatory Th-17 cells. In inflamed tissues, it is always not one but a complex “cytokine network” that leads to a specific signature and response. Protein interaction network (Figure 8) and gene-set functional enrichment analysis (Table 2) indicate that the Th-17 signaling pathway (IL-17A, RORγt, and IL-23R) significantly influences several biological processes, molecular functions, and cellular components linked to trophic, inflammatory, and tissue immune response. Defining the regulatory network by which UC-MSCs modulate Th-17 pathway may help in improving tissue repair goals. By leveraging AI and deep learning, it might be possible to forecast the efficacy of MSCs in an inflammatory setting, enhancing the effectiveness of MSC-based regenerative medicine methods and ultimately leading to successful personalized therapeutic options. While the inducing cytokine signals and core transcription factors driving the differentiation toward Th-17 lineage are well known, detailed mechanistic interactions between the key components are poorly understood [38]. In parallel, analyzing Th-17 cell differentiation dynamics using a novel integrative modeling framework for time-course RNA sequencing data may represent a novel tool to understand the Th-17 regulatory network and dependency in Th-17 polarizing conditions.

## Figures and Tables

**Table 1:** List of monoclonal antibodies for flow cytometry.

Human Primary Antibody	Species	Dilution	Source	Isotype Control
Anti-CD73-APC	Mouse	1/20	BD Biosciences (San Jose, CA, USA)	APC mouse IgG1
Anti-CD90-PE	Mouse	1/20	R&D Systems (Minneapolis, MN, USA)	PE mouse IgG2A
Anti-105-FITC	Mouse	1/20	BioLegend (San Diego, CA, USA)	FITC mouse IgG1
Anti-CD34-PC5	Mouse	1/20	BD Biosciences	PC5 mouse IgG1
Anti-CD14-PE	Mouse	1/20	BD Biosciences	PE mouse IgG2A
Anti-CD19-PE	Mouse	1/20	BD Biosciences	PE mouse IgG1
Anti-CD45-PC7	Mouse	1/20	BD Biosciences	PC7 mouse IgG2A
Anti-HLA-DR-PerCP	Mouse	1/20	BD Biosciences	PerCP mouse IgG2A

**Table 2:** Gene-set functional enrichment analysis and statistics of IL-17A, RORγt, and IL-23R network interactions and association. Gene ontology (GO) is used to perform enrichment analysis for biological processes, molecular functions, and cellular components.

Network Stats				
	Number of nodes: 21	Expected number of edges: 53		
	Number of edges: 174	PPI enrichment *p*-value: <1.0 × 10^−16^		
	Average node degree: 16.6	*Your network has significantly more interactions*		
	Avg. local clustering coefficient: 0.898	*than expected*		
**Functional enrichments in your network**				
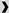	**Biological Process (Gene Ontology)**			
*GO-term*	*Description*	*Count in network*	*Strength*	*False-discovery rate*
GO-1900100	Positive regulation of plasma cell differentiation	2 of 3	2.8	0.00098
GO-0060559	Positive regulation of calcidiol 1-monooxygenase activity	2 of 3	2.8	0.00098
GO-0001660	Fever generation	2 of 3	2.8	0.00098
GO-2000340	Positive regulation of chemokine (C-X-C motif) ligand 1 production	2 of 5	2.75	1.00 × 10^−5^
GO-0032747	Positive regulation of interleukin-23 production	2 of 7	2.6	1.89 × 10^−5^
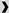	**Molecular Function (Gene Ontology)**			
*GO-term*	*Description*	*Count in network*	*Strength*	*False-discovery rate*
GO-0004920	Positive regulation of plasma cell differentiation	2 of 2	2.97	0.0020
GO-0030368	Positive regulation of calcidiol 1-monooxygenase activity	2 of 8	2.55	7.02 × 10^−5^
GO-0005149	Fever generation	2 of 17	2.04	0.0474
GO-2004896	Positive regulation of chemokine (C-X-C motif) ligand 1 production	2 of 96	1.94	1.67 × 10^−12^
GO-0070851	Positive regulation of interleukin-23 production	2 of 135	1.69	5.07 × 10^−8^
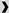	**Cellular Component (Gene Ontology)**			
*GO-term*	*Description*	*Count in network*	*Strength*	*False-discovery rate*
GO-0098802	Plasma membrane signaling receptor complex	4 of 194	2.29	0.0280
GO-0005887	Integral component of plasma membrane	9 of 1706	0.69	0.0280
GO-0005615	Extracellular space	12 of 3247	0.54	0.0280
GO-0005576	Extracellular region	14 of 4175	0.5	0.0192
**Network Stats**				
	Number of nodes: 21	Expected number of edges: 47		
	Number of edges: 177	PPI enrichment *p*-value: <1.0 × 10^−16^		
	Average node degree: 16.9	*Your network has significantly more interactions*		
	Avg. local clustering coefficient: 0.903	*than expected*		
**Functional enrichments in your network**				
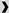	**Biological Process (Gene Ontology)**			
*GO-term*	*Description*	*Count in network*	*Strength*	*False-discovery rate*
GO-2000330	Positive regulation of T-helper 17 cell lineage commitment	4 of 4	2.97	1.08 × 10^−8^
GO-0051142	Positive regulation of NK T cell proliferation	2 of 10	2.82	1.39 × 10^−14^
GO-2000635	Negative regulation of primary miRNA processing	2 of 3	2.8	0.00068
GO-1900100	Positive regulation of plasma cell differentiation	2 of 3	2.8	0.00068
GO-0038155	Interleukin-23-mediated signaling pathway	2 of 3	2.8	0.00068
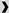	**Molecular Function (Gene Ontology)**			
*GO-term*	*Description*	*Count in network*	*Strength*	*False-discovery rate*
GO-0004920	Interleukin-10 receptor activity	2 of 2	2.97	0.0021
GO-0005143	Interleukin-12 receptor binding	2 of 3	2.8	0.0033
GO-0004904	Interferon receptor activity	2 of 5	2.57	0.0066
GO-0031702	Type 1 angiotensin receptor binding	2 of 6	2.5	0.0083
GO-0005131	Growth hormone receptor binding	2 of 11	2.23	0.0206
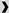	**Cellular Component (Gene Ontology)**			
*GO-term*	*Description*	*Count in network*	*Strength*	*False-discovery rate*
GO-0070743	Plasma membrane signaling receptor complex	2 of 2	2.97	0.0015
GO-0072536	Integral component of plasma membrane	6 of 7	2.91	2.32 × 10^−12^
GO-0042022	Extracellular space	5 of 7	2.83	4.41 × 10^−10^
GO-0098802	Extracellular region	9 of 194	1.64	2.94 × 10^−10^
GO-0009897	External side of plasma membrane	5 of 388	1.08	0.0098
**Network Stats**				
	Number of nodes: 21	Expected number of edges: 36		
	Number of edges: 106	PPI enrichment *p*-value: < 1.0 × 10^−16^		
	Average node degree: 10.1	*Your network has significantly more interactions*		
	Avg: local clustering coefficient: 0.713	*than expected*		
**Functional enrichments in your network**				
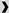	**Biological Process (Gene Ontology)**			
*GO-term*	*Description*	*Count in network*	*Strength*	*False-discovery rate*
GO-1904017	Cellular response to Thytoglobulin triiodothyronine	2 of 3	2.8	0.0014
GO-0018076	N-terminal peptidyl-lysine acetylation	2 of 3	2.8	0.0014
GO-2000340	Positive regulation of chemokine (C-X-C motif) ligand 1 production	2 of 5	2.57	0.0024
GO-1904179	Positive regulation of adipose tissue development	2 of 8	2.55	3.83 × 10^−5^
GO-0035357	Peroxisome proliferator activated receptor signaling pathway	2 of 9	2.5	4.82 × 10^−5^
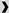	**Molecular Function (Gene Ontology)**			
*GO-term*	*Description*	*Count in network*	*Strength*	*False-discovery rate*
GO-0017162	Aryl hydrocarbon receptor binding	3 of 9	2.5	4.88 × 10^−5^
GO-0008142	Oxysterol binding	2 of 7	2.43	0.0042
GO-0030368	interleukin-17 receptor activity	2 of 8	2.37	0.0052
GO-0046965	Nuclear retinoid X receptor binding	3 of 14	2.3	0.00014
GO-0097677	STAT family protein binding	1	2.23	0.0088
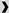	**Cellular Component (Gene Ontology)**			
*GO-term*	*Description*	*Count in network*	*Strength*	*False-discovery rate*
GO-0017053	Transcription repressor complex	3 of 77	1.56	0.0111
GO-0000118	Histone deacetylase comples	3 of 80	1.55	0.0116
GO-0090575	RNA polymerase II transcription regulator complex	6 of 256	1.34	8.13 × 10^−5^
GO-0005667	Transcription regulator complex	11 of 517	1.3	2.59 × 10^−9^
GO-0000785	Chromatin	13 of 1285	0.98	5.20 × 10^−8^

## Data Availability

The data presented in this study might be available depending on the type of demand and use and are linked to the authorities’ authorization. A request must be sent to the corresponding author with the permission of all authors.

## Supplementary Materials

The following supporting information can be downloaded at: https://www.mdpi.com/article/10.3390/cells13020169/s1, Figure S1: Array profile of cytokines related to Th-17 pathway. In normal and inflammatory setting, UC-MSCs (*n* = 5) were cocultured at a (1:80) cell ratio with either nonstimulated or PHA/IL-2-stimulated T cells, as indicated. The levels of the various cytokines were determined as previously described in the Materials and Methods section. The data for IL-1β, IL-4, IL-6, IL-10, IL-17A, IL-17F, IL-21, IL-22, IL-23, IL-25, IL-31, IL-33, INF-γ, sCD40, and TNF-α are shown as concentration ± SEM (pg/mL). * *p* ≤ 0.05, ** *p* ≤ 0.01, *** *p* ≤ 0.001 compared to the matching control. UC-MSCs: umbilical cord mesenchymal stromal / stem cells; UC-MSCs^INF^: preconditioned umbilical cord mesenchymal stromal / stem cells; NST: nonstimulated T cells; ST: PHA/IL-2-stimulated T cells.
