# Supplementary material for: Umbilical Cord Mesenchymal Stromal/Stem Cells and Their Interplay with Th-17 Cell Response Pathway
Source: Cells. 2024 Jan 16;13(2):169. doi: 10.3390/cells13020169 (PMC10814115; doi:10.3390/cells13020169)
Supplement: Supplementary file 1 [file cells-13-00169-s001.zip › cells-2746168-supplementary.pdf]

Supplementary Figure S1

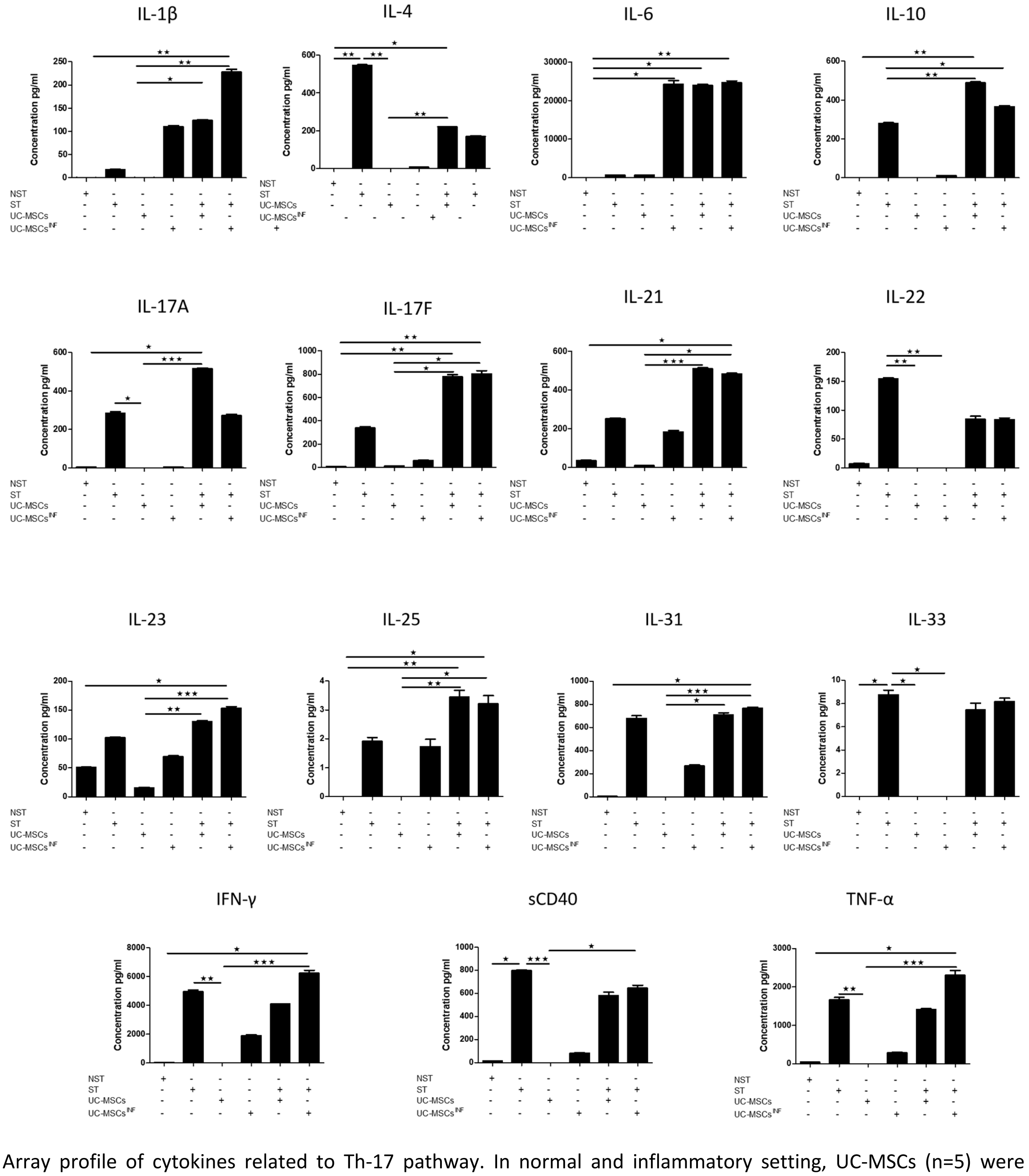

Array profile of cytokines related to Th-17 pathway. In normal and inflammatory setting, UC-MSCs (n=5) were cocultured at a (1:80) cell ratio with either nonstimulated or PHA/IL-2-stimulated T cells, as indicated. The levels of the various cytokines were determined as previously described in the Materials and Methods section. The data for IL-1β, IL-4, IL-6, IL-10, IL-17A, IL-17F, IL-21, IL-22, IL-23, IL-25, IL-31, IL-33, INF-γ, sCD40, and TNF-α are shown as concentration ± SEM (pg/mL). * p 0.05, ** p 0.01, *** p 0.01 compared to the matching control. UC-MSCs: umbilical cord mesenchymal stromal / stem cells; UC-MSCs^{INF}: preconditioned umbilical cord mesenchymal stromal / stem cells; NST: nonstimulated T cells; ST: PHA/IL-2-stimulated T cells.
